# Supplementary material for: Role of Dietary Defatted Rice Bran in the Modulation of Gut Microbiota in AOM/DSS-Induced Colitis-Associated Colorectal Cancer Rat Model
Source: Nutrients. 2023 Mar 22;15(6):1528. doi: 10.3390/nu15061528 (PMC10052090; doi:10.3390/nu15061528)
Supplement: Supplementary file 1 [file nutrients-15-01528-s001.zip › nutrients-2276468-supplementary.pdf]

Table S1. Effect of defatted rice bran on body weight change (%) (mean ± S.E.M.).

Week	Experimental group					
	Control (n=10)	DRBL (n=10)	DRBH (n=10)	Induction (n=12)	Induction + DRBL (n=12)	Induction + DRBH (n=12)
1	65.31 ± 5.68	65.98 ± 3.75	65.16 ± 4.76	69.61 ± 3.97	67.81 ± 3.66	69.79 ± 4.48
2	115.88 ± 7.79	114.61 ± 5.51	112.09 ± 6.49	119.99 ± 5.00	117.48 ± 5.25	114.77 ± 6.00
3	175.37 ± 10.73	169.10 ± 8.23	168.77 ± 8.72	175.92 ± 5.93	171.93 ± 5.61	171.00 ± 7.35
4	228.62 ± 13.93	222.59 ± 11.05	220.52 ± 10.94	217.61 ± 7.65	214.51 ± 7.17	214.74 ± 8.53
5	274.13 ± 16.13	279.09 ± 16.48	260.53 ± 13.45	239.63 ± 8.96	242.53 ± 9.77	247.40 ± 10.16
6	309.73 ± 18.86	296.70 ± 13.61	294.11 ± 15.30	236.49 ± 11.63*	245.97 ± 11.83*	256.68 ± 12.57*
7	338.99 ± 19.61	328.11 ± 16.72	321.93 ± 17.32	295.20 ± 13.22*	299.08 ± 13.24*	302.75 ± 12.59*
8	362.07 ± 21.62	355.31 ± 16.71	349.30 ± 18.69	274.95 ± 11.85*	280.11 ± 17.09*	290.18 ± 11.66*
9	382.62 ± 23.65	376.60 ± 18.17	368.90 ± 20.08	329.39 ± 15.20	329.69 ± 16.99	328.92 ± 15.19
10	401.59 ± 24.51	396.89 ± 20.16	387.29 ± 22.17	348.36 ± 14.31	352.13 ± 15.92	355.89 ± 15.13
11	413.06 ± 25.76	411.38 ± 21.15	395.44 ± 24.62	368.98 ± 16.27	371.19 ± 16.22	372.01 ± 15.54
12	425.31 ± 25.57	423.96 ± 21.38	411.80 ± 22.16	379.68 ± 14.94	383.06 ± 15.50	382.44 ± 14.80
13	434.13 ± 26.29	435.77 ± 21.87	423.43 ± 22.03	388.10 ± 16.42	390.43 ± 17.52	393.80 ± 15.61

Data from all experimental groups are compared using one-way ANOVA followed by Tukey's HSD post-hoc test. Mean with an asterisk (*) superscript in each column is significantly different ($p < 0.05$) when compared to the control group.

Table S2. Effect of defatted rice bran on food intake (g) (mean ± S.E.M.).

Week	Experimental group					
	Control (n=10)	DRBL (n=10)	DRBH (n=10)	Induction (n=12)	Induction + DRBL (n=12)	Induction + DRBH (n=12)
1	19.94 ± 0.25	20.86 ± 0.54	20.45 ± 0.51	19.08 ± 0.46	18.97 ± 0.60	19.20 ± 0.44
2	21.60 ± 0.63	22.34 ± 0.55	21.42 ± 0.74	21.60 ± 0.43	21.22 ± 0.55	20.68 ± 0.73
3	25.48 ± 0.32	24.92 ± 0.55	24.78 ± 0.35	23.43 ± 0.36*	23.17 ± 0.25*	21.37 ± 0.94*
4	20.34 ± 1.64	20.50 ± 1.93	21.94 ± 1.21	22.75 ± 1.31	20.53 ± 1.23	20.10 ± 1.31
5	26.32 ± 0.48	24.90 ± 0.56	22.66 ± 0.73	21.70 ± 0.58*	21.95 ± 0.50*	23.50 ± 0.83*
6	25.20 ± 0.70	24.90 ± 0.31	23.46 ± 0.51	18.17 ± 1.08*	16.98 ± 0.52*	18.82 ± 0.50*
7	23.78 ± 0.79	22.74 ± 0.66	20.80 ± 1.01	24.15 ± 0.66	22.95 ± 0.65	21.07 ± 0.84#
8	22.86 ± 1.50	24.16 ± 1.09	22.14 ± 0.61	18.60 ± 1.05*	14.35 ± 1.94*	14.15 ± 1.38*
9	26.06 ± 0.32	22.42 ± 1.27	22.00 ± 0.98	25.22 ± 0.94	23.98 ± 0.65	23.75 ± 0.79
10	24.62 ± 0.42	22.16 ± 0.69	21.42 ± 0.38	23.33 ± 0.67	20.34 ± 1.09*	21.58 ± 0.75*
11	22.02 ± 0.84	19.48 ± 0.97	21.42 ± 0.96	23.67 ± 0.59	19.90 ± 1.16#	18.75 ± 0.72#
12	22.78 ± 0.34	21.44 ± 0.67	19.52 ± 0.72	22.83 ± 0.69	20.83 ± 1.01	21.27 ± 0.77
13	21.24 ± 0.80	21.26 ± 0.77	20.52 ± 0.65	20.93 ± 0.84	18.58 ± 0.76*#	16.67 ± 0.83*#

Data from all experimental groups are compared using one-way ANOVA followed by Tukey's HSD post-hoc test. Mean with an asterisk (*) and number signs (#) superscript in each column is significantly different ($p < 0.05$) when compared to the control and induction groups, respectively.

Table S3. Effect of defatted rice bran on colon weight (g), length (cm), and relative colonic weight/length ratio (g/cm) in experimental group (mean ± S.E.M.).

Parameters	Experimental group					
	**Control (n=10)**	**DRBL (n=10)**	**DRBH (n=10)**	**Induction (n=12)**	**Induction + DRBL (n=12)**	**Induction + DRBH (n=12)**
Colon weight (g)	1.33 ± 0.17	1.33 ± 0.11	1.34 ± 0.18	1.40 ± 0.18	1.32 ± 0.23	1.35 ± 0.24
Colon length (cm)	20.45 ± 1.44	21.27 ± 2.31	20.57 ± 2.28	17.89 ± 1.07	18.39 ± 1.13	18.70 ± 2.17
Relative colon weight/length ratio (g/cm)	0.065 ± 0.007	0.063 ± 0.008	0.066 ± 0.008	0.078 ± 0.007*	0.070 ± 0.010	0.072 ± 0.009

Data from all experimental groups are compared using one-way ANOVA followed by Tukey's HSD post-hoc test. Mean with an asterisk (*) superscript in each column is significantly different ($p < 0.05$) when compared to the control group.

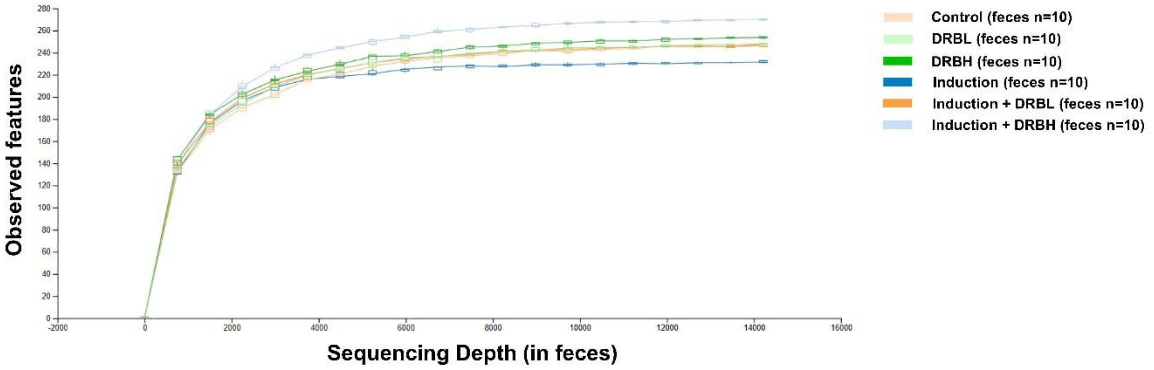

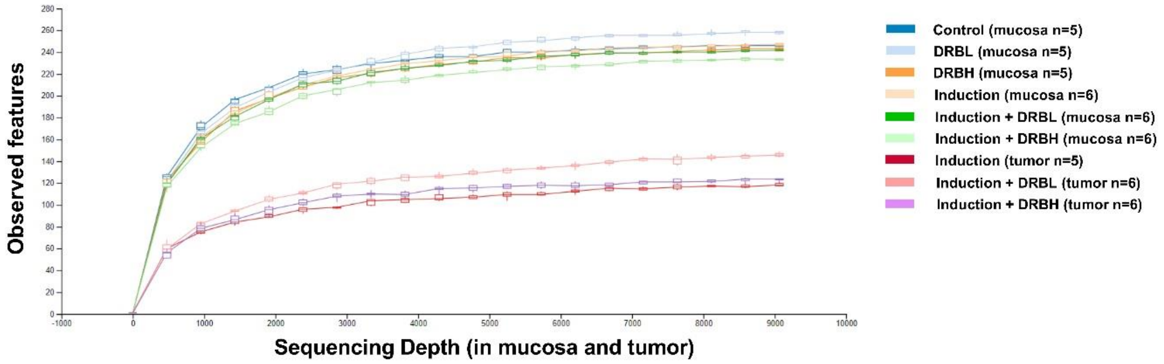

Figure S1. The rare fraction curves of alpha diversity in colonic feces, mucosa, and tumors.

Table S4. Comparison of relative abundance (%) of bacterial phylum in feces.

Phylum	Experimental group (in feces)					
	**Control (n=10)**	**DRBL (n=10)**	**DRBH (n=10)**	**Induction (n=12)**	**Induction + DRBL (n=12)**	**Induction + DRBH (n=12)**
Firmicutes	58.76	69.27	64.78	63.08	68.66	63.66
Verrucomicrobia	23.02	17.76	14.48	20.25	16.29	19.87
Bacteroidetes	15.75	11.15	18.59	14.58	13.06	14.40
Actinobacteria	1.84	1.42	1.50	1.43	1.54	1.46
Proteobacteria	0.48	0.29	0.53	0.52	0.28	0.44
Patescibacteria	0.15	0.11	0.12	0.13	0.17	0.17

Table S5. Comparison of relative abundance (%) of bacterial family in feces.

Family	Experimental group (in feces)					
	**Control (n=10)**	**DRBL (n=10)**	**DRBH (n=10)**	**Induction (n=12)**	**Induction + DRBL (n=12)**	**Induction + DRBH (n=12)**
Lachnospiraceae	18.66	23.54	24.95	21.19	25.24	23.09
Akkermansiaceae	23.02	17.76	14.48	20.25	16.29	19.87
Peptostreptococcaceae	10.66	15.85	10.42	12.69	12.10	11.94
Lactobacillaceae	10.05	8.32	8.39	6.99	9.09	8.86
Muribaculaceae	10.23	6.94	10.21	8.94	7.41	7.70
Oscillospiraceae	2.93	4.20	5.74	4.48	5.13	4.74
Erysipelotrichaceae	4.10	4.30	3.10	5.02	4.26	3.35
Ruminococcaceae	4.43	4.30	5.14	2.96	3.77	3.66
Prevotellaceae	3.04	2.48	5.38	3.21	3.71	4.67
Clostridiaceae	2.46	2.98	0.98	4.02	3.15	2.19
[Eubacterium] coprostanoligenes group	1.93	2.02	2.16	1.92	2.29	2.30
Eggerthellaceae	1.66	1.33	1.41	1.29	1.45	1.38
Bacteroidaceae	1.41	0.97	1.76	1.38	1.15	1.25
Rikenellaceae	1.07	0.75	1.24	1.05	0.78	0.78

Table S6. Comparison of relative abundance (%) of bacterial genus in feces.

Genus	Experimental group (in feces)					
	Control (n=10)	DRBL (n=10)	DRBH (n=10)	Induction (n=12)	Induction + DRBL (n=12)	Induction + DRBH (n=12)
Akkermansia	23.019	17.760	14.479	20.254	16.295	19.873
Lactobacillus	10.053	8.317	8.394	6.986	9.092	8.858
Alloprevotella	2.402	1.688	3.749	2.601	2.779	3.045
Prevotellaceae UCG-001	0.636	0.795	1.626	0.610	0.928	1.624
Ruminococcus	0.814	0.731	1.305	0.523	0.825	0.983
Ruminococcaceae	0.034	0.146	0.129	0.020	0.075	0.142
Butyricicoccus	0.018	0.018	0.034	0.019	0.033	0.028
Roseburia	0.013	0.037	0.058	0.055	0.043	0.053
Turicibacter	3.498	3.737	2.982	4.061	4.078	3.116
Clostridium sensu stricto 1	2.458	2.977	0.984	4.016	3.146	2.185
Enterococcus	0.044	0.105	0.085	0.291	0.182	0.264
Escherichia-Shigella	0.021	0.024	0.014	0.238	0.056	0.148
Citrobacter	0.000	0.000	0.000	0.014	0.007	0.009

Table S7. Comparison of relative abundance (%) of bacterial phylum in colonic mucosa and tumor.

Phylum	Experimental group (in mucosa)						Experimental group (in tumor)		
	Control (n=5)	DRBL (n=5)	DRBH (n=5)	Induction (n=6)	Induction + DRBL (n=6)	Induction + DRBH (n=6)	Induction (n=5)	Induction + DRBL (n=6)	Induction + DRBH (n=6)
Firmicutes	55.45	57.91	51.56	54.50	56.88	55.63	48.77	66.90	52.99
Bacteroidetes	24.64	18.38	27.95	17.74	22.60	22.96	6.89	8.17	6.52
Verrucomicrobia	11.32	18.90	11.86	11.05	9.03	12.86	5.48	4.23	5.98
Proteobacteria	5.49	2.82	6.13	15.48	9.36	6.50	37.31	19.68	31.56
Actinobacteria	2.99	1.87	2.29	1.17	1.98	1.97	1.37	0.93	2.94
Patescibacteria	0.06	0.11	0.11	0.06	0.13	0.07	0.02	0.08	0.01

Table S8. Comparison of relative abundance (%) of bacterial family in colonic mucosa and tumor.

Family	Experimental group (in mucosa)						Experimental group (in tumor)		
	Control (n=5)	DRBL (n=5)	DRBH (n=5)	Induction (n=6)	Induction + DRBL (n=6)	Induction + DRBH (n=6)	Induction (n=5)	Induction + DRBL (n=6)	Induction + DRBH (n=6)
Lachnospiraceae	17.97	18.47	18.29	17.53	14.07	17.55	5.76	6.74	4.40
Muribaculaceae	15.37	11.13	15.85	10.06	13.15	11.87	4.07	4.07	2.57
Ruminococcaceae	14.67	15.81	16.86	12.06	12.49	14.76	5.85	6.52	6.21
Akkermansiaceae	11.32	18.90	11.86	11.05	9.03	12.86	5.48	4.23	5.98
Lactobacillaceae	9.33	7.33	6.11	5.07	8.47	7.19	18.32	27.92	7.24
Prevotellaceae	6.71	4.92	8.18	3.91	5.85	6.82	1.76	2.01	1.94
Peptostreptococcaceae	6.21	7.89	5.38	6.96	7.67	4.69	2.72	3.50	1.19
Erysipelotrichaceae	3.54	3.08	1.71	2.88	3.36	2.11	1.22	1.62	0.55
Caulobacteraceae	2.94	1.45	4.40	1.84	0.19	0.27	10.20	6.29	3.83
Eggerthellaceae	2.64	1.64	1.95	1.04	1.80	1.81	0.94	0.56	2.71
Clostridiaceae 1	1.62	2.44	0.53	2.02	1.40	1.04	0.44	0.78	0.30
Bacteroidaceae	1.59	1.26	2.18	2.76	2.52	3.34	0.61	1.40	1.78
Burkholderiaceae	1.46	0.76	0.91	0.47	0.39	0.59	0.18	0.20	0.03
Rikenellaceae	0.95	1.02	1.58	0.97	1.08	0.88	0.36	0.68	0.21
Clostridiales vadinBB60 group	0.71	1.17	1.01	1.19	1.38	1.11	0.72	0.66	0.33

Table S9. Comparison of relative abundance (%) of bacterial genus in colonic mucosa and tumor.

Genus	Experimental group (in mucosa)						Experimental group (in tumor)		
	Control (n=5)	DRBL (n=5)	DRBH (n=5)	Induction (n=6)	Induction + DRBL (n=6)	Induction + DRBH (n=6)	Induction (n=5)	Induction + DRBL (n=6)	Induction + DRBH (n=6)
Lactobacillus	9.327	7.328	6.108	5.066	8.471	7.186	18.322	27.916	20.237
Akkermansia	11.324	18.899	11.856	11.051	9.034	12.860	5.480	4.227	5.977
Alloprevotella	5.613	3.517	5.761	3.256	4.192	4.141	1.289	1.410	0.929
Prevotellaceae UCG-001	1.015	1.364	2.319	0.634	1.649	2.670	0.272	0.524	0.982
Ruminococcus	0.373	0.554	0.741	0.362	0.502	0.688	0.235	0.259	0.172
Roseburia	0.232	0.801	1.070	0.557	0.171	0.506	0.175	0.195	0.132
Butyricicoccus	0.159	0.088	0.190	0.077	0.118	0.123	0.158	0.050	0.040
Enterococcus	0.104	0.203	0.057	5.472	6.883	6.164	12.693	18.583	24.232
Escherichia-Shigella	0.062	0.038	0.128	6.502	3.953	3.407	12.531	8.409	6.121
Citrobacter	0.013	0.013	0.000	4.358	3.280	1.274	12.892	2.754	9.598
Clostridium sensu stricto 1	1.562	2.445	0.525	2.001	1.399	1.039	0.430	0.719	0.296
Mycobacterium	0.000	0.000	0.000	0.004	0.000	0.000	0.055	0.037	0.031
